# Data for non-invasive (photo) individual fish identification of multiple species

**DOI:** 10.1016/j.dib.2023.109221

**Published:** 2023-05-09

**Authors:** Dinara Bartunek, Petr Cisar

**Affiliations:** Laboratory of Signal and Image Processing, Institute of Complex Systems, Faculty of Fisheries and Protection of Waters, CENAKVA, University of South Bohemia in Ceske Budejovice, Zámek 136, Nové Hrady 373 33, Czechia

**Keywords:** Fish lateral image, Automation, Machine learning, Computer vision, Fish individual identification, Non-invasive identification, Tagging

## Abstract

This paper describes data from five studies focused on the individual fish identification of the same species. The lateral images of five fish species are present in the dataset. The dataset's primary purpose is to provide a data to develop a non-invasive and remote method of individual fish identification using fish skin patterns, which can serve as a substitute for the common invasive fish tagging. The lateral images of the whole fish body on the homogenous background for Sumatra barb, Atlantic salmon, Sea bass, Common carp and Rainbow trout are available with automatically extracted parts of the fish with skin patterns. A different number of individuals (Sumatra barb – 43, Atlantic salmon – 330, Sea bass – 300, Common carp – 32, Rainbow trout - 1849) were photographed by the digital camera Nikon D60 under controlled conditions. The photographs of only one side of the fish with several (from 3 to 20) repetitions were taken. Common carp, Rainbow trout and Sea bass were photographed out of the water. Atlantic salmon was photographed underwater, out of the water, and the eye of the fish was photographed by the microscope camera. Sumatra barb was photographed under the water only. For all species, except Rainbow trout, the data collection was repeated after a different period (Sumatra barb – four months, Atlantic salmon – six months, Sea bass – one month, Common carp – four months) to collect the data for a study of skin patter changes (ageing). The development of the method for photo-based individual fish identification was performed on all datasets. The identification accuracy for all species for all periods was 100% using the nearest neighbour classification. Different methods for skin pattern parametrization were used.

The dataset can be used to develop remote and non-invasive individual fish identification methods. The studies focused on the discrimination power of the skin pattern can benefit from it. The changes of skin patterns due to fish ageing can be explored from the dataset.

**Specifications Table**

**Table:** 

Subject	Computer Vision and Pattern Recognition
Specific subject area	Individual identification; object detection, pattern parametrization
type of data	Colour image; code for data processing
How the data were acquired	Data were collected with the digital camera Nikon D60. Images of eye of the fish were collected with micro camera DinoCapture. MatLab R2020b was used for data pre-processing.
Data format	Raw (nef images) and processed data (png images)
Description of data collection	Data were collected under controlled conditions. The white light, photo tent and green homogenous background were used during data collection. The lateral images of the whole fish body on the homogenous background for Sumatra barb, Atlantic salmon, Sea bass, Common carp and Rainbow trout are available with automatically extracted parts of the fish with skin patterns. A different number of individuals (Sumatra barb – 43, Atlantic salmon – 330, Sea bass – 300, Common carp – 32, Rainbow trout - 1849) were photographed by the digital camera Nikon D60 under controlled conditions. The photographs of only one side of the fish with several (from 3 to 20) repetitions were taken. Common carp, Rainbow trout and Sea bass were photographed out of the water. Atlantic salmon was photographed underwater, out of the water, and the eye of the fish was photographed by the microscope camera. Sumatra barb was photographed under the water only. For all species, except Rainbow trout, the data collection was repeated after a different period (Sumatra barb – four months, Atlantic salmon – six months, Sea bass – one month, Common carp – four months) to collect the data for a study of skin patter changes (ageing). The fish were anaesthetized for the data collection. The only exception is for rainbow trout. Fish was killed for data collection.
Data source location	Atlantic Salmon - NOFIMA a.s., Sjølsengvegen 22, 6600 Sunndalsøra, Norway Rainbow trout, Common carp and Sumatra barb – USB, Zámek 136, Nové Hrady, Czech republic Sea bass – HCMR, 71003 Heraklion, Crete, Greece
Data accessibility	The data (original and processed) and protocols describing the data collection process and preprocessing, are stored in the Zenodo repository. Totally five species were collected. Only rainbow trout dataset will be available after publishing the results. Non - invasive identication of individuals of European seabass Dicentrarchus labrax (short term). DOI:10.5281/zenodo.7517321 https://doi.org/10.5281/zenodo.7517321 Long term photo - identification of european seabass. DOI: 10.5281/zenodo.7665241 https://doi.org/10.5281/zenodo.7665241 Common carp individual identification. DOI: 10.5281/zenodo.7654255 https://doi.org/10.5281/zenodo.7654255 Photo - Identification of individuals of Atlantic salmon (eye dataset). DOI: https://doi.org/10.5281/zenodo.7654844
	https://doi.org/10.5281/zenodo.7654844 Non-invasive identification of individuals of Atlantic salmon (tent data). DOI: 10.5281/zenodo.7690930 https://doi.org/10.5281/zenodo.7690930 Non - invasive identification of individuals of Atlantic salmon (aquarium data). DOI: 10.5281/zenodo.7674791 https://doi.org/10.5281/zenodo.7674791 Non - invasive identification of individuals of Sumatra barb Puntigrus tetrazona. DOI: 10.5281/zenodo.7517085 https://doi.org/10.5281/zenodo.7517085 Also, Here is the link to GIT hub repository of codes which were used in our studies: PetrCisar/Fish-identification: Public release. DOI: 10.5281/zenodo.7762305 https://zenodo.org/badge/latestdoi/609022973
Related research article	D. Bekkozhayeva, P. Cisar, Image-Based Automatic Individual Identification of Fish without Obvious Patterns on the Body (Scale Pattern), Appl. Sci. 12 (2022) 5401. https://doi.org/10.3390/app12115401.

## Value of the Data

• The dataset is the largest (the highest number of fish and the longest period of data collection) data collection suitable for the development and testing of the methods for individual fish identification. No similar dataset exists. This data could be useful to the researchers who deal with the identification of fish individuals welfare indicators and disease monitoring systems.
• The computer vision specialist and fish biologist can benefit from the data.
• The data can be mainly used by the developers to create an automatic system for individual fish identification.
• The discrimination power of different skin patterns can also be studied due to the high number of individual fish.
• The skin pattern changes due to the ageing can be studied because the dataset contains long-term data collection for Atlantic salmon.

## 1 Objective

The reason for creating this dataset was the non-existence of a freely accessible dataset useful for individual fish identification and to study the discrimination power of fish skin patterns. Several studies on fish identification based on fish skin patterns exist, but the data are not accessible and it was designed mainly for human-based processing. This dataset contains the images of five fish species, a high number of individuals and long-term data collections.

## 2 Data Description

We collected a large dataset of five fish species for the development and testing of the methods for individual fish identification of the same species. Those data sets were used for the development of our own approaches for the identification of fish individuals. The basic description of the datasets is presented in Table 1. Table 2 contains information about collected raw images of the fish, and Table 3 summarizes automatically extracted fish skin patterns (ROI) for identification. The data are grouped based on the fish species. For each data collection and data processing, the protocol in Zenodo system exists. This protocol describes experimental conditions/data processing and contains relevant images. All codes published in Zenodo system [1].

**Table 1:** General information about the dataset with recommendation for data usage.

Species	Description	Pattern parametrization method	Reference to the paper
Sumatra barb dataset	A small number of fish, underwater, two data collections in two weeks. Suitable for first testing of identification.	HOG	[2]
Atlantic salmon dataset (tent dataset)	An intermediate of fish, out of the water, four data collections in six months. Suitable for a test of pattern changes due to ageing.	HOG, mutual dot position	[3]
Atlantic salmon dataset (aquarium dataset)	An intermediate number of fish, underwater, four data collections in six months. Suitable for a test of the influence of the underwater data collection on the identification.		
Atlantic salmon dataset (fish eye dataset)	An intermediate number of fish, out-of-water eye images, four data collections in six months. Suitable for a test of the specific fish eye pattern.	Wavelet	[4]
European seabass dataset	An intermediate number of fish, out-of-water, two data collection in two months. Suitable for a fish identification test without an obvious pattern but with a lateral line.	HOG	[5]
Common carp dataset	A small number of fish, out-of-water, four data collections in four months. Suitable for a test of pattern changes due to the ageing of the fish without an obvious skin pattern.	HOG	
Rainbow trout	A high number of fish, out-of-water, one data collection. Suitable for a test of pattern discriminative power of the skin pattern.	HOG	Not yet published.

**Table 2:** Details of raw collected images for fish species.

Fish species	Image resolution	N of the fish	Data collection conditions	The best obtained result Accuracy,%
Sumatra barb dataset	2393×1701 pixel, with 24 bits/pixel and three colour channels	43	Aquarium	100
Atlantic salmon dataset (tent dataset)	5616×3744 pixel, with 12 bits/pixel and three colour channels	328	Out of the water (tent)	100
Atlantic salmon dataset (aquarium dataset)			Aquarium	100
Atlantic salmon dataset (fish eye dataset)	1280×1024 pixel, with 24 bits/pixel and three colour channels		Microscope camera out of the water	100
European seabass dataset	4288×2848 pixel, with 12 bits/pixel and three colour channels	300	Out of the water	100
Common carp dataset	4310×2868 pixel, with 32 bits/pixel and three colour channels	32	Out of the water	100
Rainbow trout	4288×2848 pixel, with 32 bits/pixel and three colour channel	1602	Out of the water	100

**Table 3:** Extracted patterns (ROI) for individual fish species.

Specie	Name of the ROI	Description
Sumarta barb	ROI1	A central stripe on the body
	ROI2	Two middle stripes – narrow
	ROIW	Two middle stripes – wide
	ROIH	From head to tail – narrow

Atlantic salmon	ROI	Manual localization of dots on ROI
	eye	The tight rectangle around the eye

European seabass	ROI(LL)	Lateral line and nearest neighbourhood
	ROI(HLL)	Half of the length of ROI(LL)
	ROI(S)	A region with scales only
	ROI(O)	An operculum part

Common carp	ROI1	Middle body part (only the scales)
	ROI2	ROI1 but longer and wider
	ROI3	the operculum part

Rainbow trout	ROI	Middle body part

Dataset of five fish species was collected: Atlantic salmon *Salmo salar*, Sumatra barb *Puntigrus tetrazona*, European seabass *Dicentrarchus labrax*, common carp *Cyprinus carpio* and rainbow trout *Oncorhynchus mykiss*. Sumatra barb data and common carp datasets has one protocol each. European seabass data devided into two protocol based on a experiment types: short term and lond term datasets. Atlantic salmon datasets devided into three protocols by the data types: eye dataset, tent dataset and aquarium dataset.

## 3 Experimental Design, Materials and Methods

The digital camera Nikon D90 was used for data collection of lateral images of all fish species. The microscope camera Dino-Lite AM3113T was used to collect Atlantic salmon and Sea bass eye images. Lateral images of the fish with the head orientated to the left were photographed. Different fish rotations and positions in the image were used to provide a rich dataset. The camera was perpendicular to the fish plane. The green homogenous background and controlled white light were used to simplify automatic fish detection. All fish were anaesthetized using clove oil for data collection. The fish images were saved in RAW format to keep the highest data quality.

Different regions (ROI) of the fish body were automatically extracted for different species as a pattern for individual identification. The extraction is based on the detection of the green background using the know background colour. The largest object is then detected in the background as the fish. The ROI is then extracted based on the height and width of the detected fish to keep the same ROI position on the fish's body. The ROI is saved in PNG format in the dataset.

## 4 Atlantic Salmon [6], [7], [8]

A total of 328 fish were used for the first data collection, with an average length of 29.5 ± 2.5 cm. Thirty of the fish were tagged with PIT tags and used for the next three data collections performed over six months at two months intervals. Three types of data were taken in each session: lateral view images of the fish out of the water (in a photographic tent) and underwater (in a small aquarium) and iris of the fisheye. On average, eight images of each fish were photographed. Three ROIs were extracted from the images. A central part of the fish body was extracted from underwater images and out-of-water images. The bounding box of the eye was extracted from microscope images.

## 5 Sumatra Barb [9]

43 fish individuals of Sumatra barb were used for imaging, with an average size of 41 mm. Data were collected two times during two weeks for fish inside the aquarium. Tree images of each individual were taken in every data collection. Four ROIs were extracted for identification.

## 6 European Seabass [10], [11]

Totally 300 sea bass were used for the first data collection. The initial fish size was 15 – 17 cm for approximately one-year old fish. Ten images of each fish were collected. Randomly selected 32 fish individuals out of the 300 fish were tagged by PIT tags for second data collection. The second data collection was performed in two months. Four different ROI (regions of interest) were used for identification.

## 7 Common Carp [12]

Four data collections were performed over the four-month. Thirty-two fish individuals were used. Up to ten images were collected per each fish with an average length of 42 cm. The age of the carp was approximately two years. Three ROIs were extracted for identification.

## 8 Rainbow Trout

**Fig. 1:**
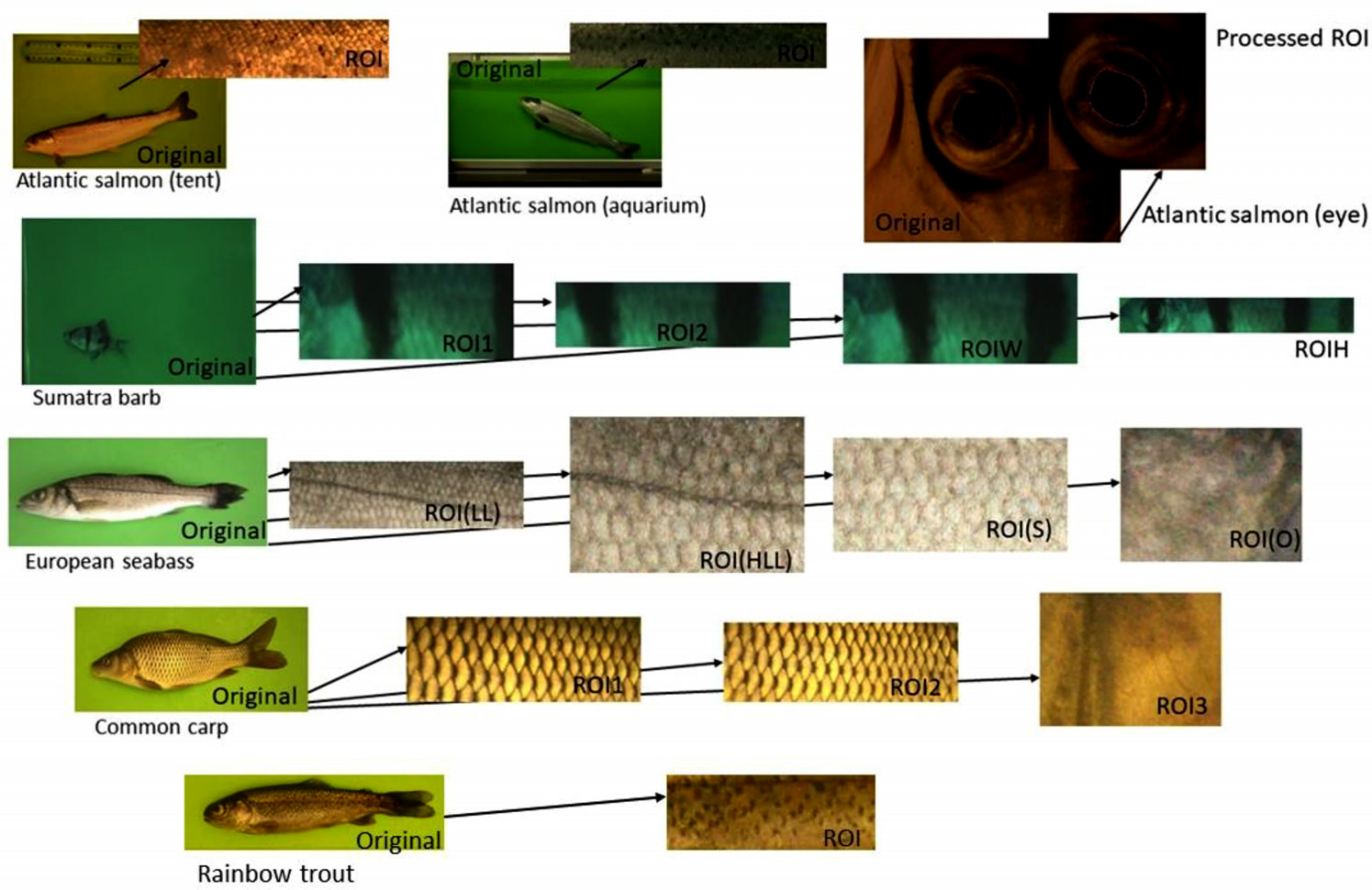
The database examples. Original data and extracted ROI of five fish species.

one data collection was done for 1849 fish individuals of rainbow trout. The size of the fish was around 7–10 cm. Fifteen fish individuals were photographed together. The image was automatically divided into sub-images containing individual fish. Three images of each fish were taken at different angles. One ROI was extracted for identification (Fig. 1).

The individual identification experiments were performed for all species. The results of those experiments are fully presented in our papers. The datasets are scaled and focused on the different aspects of fish identification. The first dataset contains images of Sumatra barb. The identification methods can be first tested on this dataset because of the obvious stripe pattern on the fish and data collection close to the real conditions. We reached the classification accuracy of 100% [2]. The long-term patterns stability was tested on Atlantic salmon data on a higher number of individuals. We reported 100% identification accuracy using specific skin dot parametrization [3]. The possibility of using a fish eye pattern was also tested on this dataset [4] with lower accuracy (81%) than for the skin dot pattern. The identification of the fish without an obvious pattern was tested on Sea bass and Common carp dataset. In both cases, we reached 100% accuracy [5]. The identification of the largest number of fish (1602 individuals) was done for Rainbow trout. The discrimination power of the pattern was tested for fish numbers close to commercial fish production. The identification accuracy was 100%.

## Ethics Statements

The work did not involve the use of human subjects, animal experiments, nor data collected from social media platforms. The fish anaesthesia and fish tagging were done according to the standard operating procedures and regulations of the infrastructure where the data were collected. The animal experiments complied with the EU Directive 2010/63/EU for animal experiments.

## CRediT authorship contribution statement

**Dinara Bartunek:** Data curation, Writing – original draft, Visualization, Investigation. **Petr Cisar:** Methodology, Conceptualization, Methodology, Supervision, Writing – review & editing.

## Declaration of Competing Interest

The authors declare that they have no known competing financial interests or personal relationships that could have appeared to influence the work reported in this paper.

## Data Availability

Non - invasive identification of individuals of Sumatra barb Puntigrus tetrazona (Original data) (Zenodo).
